# Nonylphenol, Octyphenol, and Bisphenol A in Groundwaters as a Result of Agronomic Practices

**DOI:** 10.1100/tsw.2002.219

**Published:** 2002-04-24

**Authors:** Sílvia Lacorte, Anna Latorre, Míriam Guillamon, Damià Barceló

**Affiliations:** Department of Environmental Chemistry, IIQAB-CSIC, Jordi Girona 18-26, 08034 Barcelona, Spain

**Keywords:** formulations, groundwater, pesticides, surfactants

## Abstract

Due to increasing concern about the assessment and protection of the quality of groundwater as a limited natural resource, an extensive monitoring program has been carried out, covering a highly agricultural area of Catalonia where different types of crops have been grown during the last 5–10 years. The paper reports the analytical protocol used (sampling, filtration, preconcentration, and analysis) and the most relevant organic compounds present in groundwaters from agricultural areas as well as the concentration levels found.

## INTRODUCTION

Pollutants in the watershed can leak down into the groundwater, causing contamination that is difficult and often impossible to identify and remediate[1]. Organic compounds have been detected in groundwaters as a result of contamination episodes originated from infiltration of industrial runoff, wastewater disposal, landfills, or agricultural activities[2,3]. By far, agriculture is the main source of the introduction of pesticides into the environment, where pesticide treatments are performed on a large scale, normally from helicopters or planes, or locally, by manual spraying. In all cases, pesticides are applied as a formulated product, which is a commercially available mixture of chemicals used to kill, repel, or otherwise control one or more specific pests. A formulation consists of the active ingredient(s), present at 20–30% p/v, and the inert ingredient(s), which are added to formulations to help pesticide dissolve in water, to enhance dispersion, or to improve stability. Unless inert ingredients are determined to be highly toxic, the label does not detail the identification of the specific components and their percentages. One of the most highly used pesticides additives is alkylphenol ethoxylate (APEO), like nonylphenol or octylphenol ethoxylates (NPEO and OPEO). Depending on the type of crop and the pest to be eliminated, several different types of formulated pesticides are available in which active ingredients and adjuvants are present in various amounts. In many studies, as a result of pesticide formulations applied as well as due to the use of sewage sludge as fertilizer, pesticides and other compounds have been detected in agricultural soils and groundwaters at ppb concentrations. The leaching potential and final concentration of organic contaminants in groundwater depend on their physicochemical properties as well as on the environmental conditions, e.g., the type of soil, the pH, total organic content, rain regimes, etc. However, the problem is not caused only by the pesticides found, but also by other pollutants that might be present due to the agronomical practices carried out. Therefore, we report in this paper the presence of pesticides and pesticide adjuvants in groundwaters of an intense agricultural area of Catalonia, where in general terms the amount of formulated pesticides applied annually vary from 1 to 20 kg/ha, depending on the type of crop. The objectives of the present paper were to develop a step-by-step approach for the full characterization of groundwaters. In this work, target analytes were nonylphenol and octylphenol (NP, OP), the main degradation products of APEO, and bisphenol A (BPA), which can desorb from cans and plastic bottles where pesticides are kept. The physicochemical properties of these compounds, described as persistent and with a high log Kow, make them candidate pollutants of groundwaters, especially in areas where there is a constant input of these compounds into the environment through agronomic application. In addition, these compounds have been implicated as potential endocrine disrupters (EDCs) by positive *in vitro* tests[4].

## EXPERIMENTAL METHODS/PROCEDURES

### Chemicals

NP, OP, and BPA were kindly provided by Agbar (Aigües de Barcelona). Solvents were from Merck (Darmstadt, Germany).

### Sampling

Four groundwater samples and a surface water sample were collected from an agricultural area of Catalonia (spring 2000) where vines or horticultures and fruit trees are grown. When possible, groundwater samples were pumped off the well for 10 min to purge pipes in order to get a representative sample. Otherwise, a pooled sample was obtained by gathering at least five individual samples. The sampled wells were 10–20 m deep. The TOC levels varied from 0.5 to 10 mg/l and only the dissolved phase was analyzed.

### Filtration and Extraction

Samples were taken refrigerated to the main laboratory and were filtered through 0.45-µg filters (Whatman, England). We extracted 200 ml of water sample with OASIS (n-pyrrolidone styrene divinylbenzene) solid phase extraction cartridges of 60 mg (Waters, U.S.) on a ASPEC XL (Gilson, France) instrument. Cartridges were conditioned with 6 ml of ethyl acetate, 6 ml of methanol, and 1 ml of HPLC water, and elution was carried out with ethyl acetate and methanol to a final volume of 200 µl[5].

## GC-MS Analysis

The analysis was performed with a gas chromatograph connected to a mass spectrometer Trace GC-MS instrument (Thermoquest) using a HP-5MS (30 m × 0.25 mm i.d. with 0.25-µm film thickness) containing 5% phenyl methyl siloxane (model HP 19091S-433). In all cases, mass spectrometric analysis was performed in selected ion monitoring and scan modes to ensure quantification and confirmation of the results. These protocols provided high-quality parameters with regards to linearity and dynamic range (0.01–2 µg/l), accuracy (80–97%), precision (CV < 5%), memory and interference effects, and limits of detection (< 0.01 µg/l). These conditions are optimal for the survey of organic pollutants in groundwater.

## RESULTS AND DISCUSSION

In a typical corn field, pesticides are applied at least two or three times a year at a rate of approximately 5 l/ha, which represent a pesticide input to soil of 1 kg/ha, assuming 20% of active ingredient. This application rate also supposes an input of pesticide adjuvants to soil, which can be as high as the pesticide input. The iterative application of such formulations leads to an accumulation in soil, which acts as a retention column. Once the pollutant has infiltrated into the soil, the contaminants can undergo several different mechanisms, such as adsorption, advection, biodegradation, diffusion, and dispersion, which determine their final fate. All of these processes depend on the physicochemical properties of each organic compound. In most monitoring studies, pesticides are solely analyzed in groundwaters to determine their levels as a consequence of agricultural activities. In the present study, we have monitored pesticides along with the degradation products of surfactants, mainly NP, OP, and the plastizicer BPA in four different wells. The contribution of pesticide adjuvants in a formulated pesticide can be seen in Fig. 1, which show a GC-MS chromatogram of a formulated pesticide at 2% dilution. In this case, the active ingredient was present in 33% of the formulation (peak eluting at 32 min.) while the adjuvants correspond to all peaks eluting from 10 to 21 min. In this formulated pesticide, NP, OP, and BPA were already encountered at concentrations of 8.5, 2, and 1.5 µg/l, respectively. It is for this reason that in addition to the analysis of herbicides, as active ingredients of the formulates applied, pesticide adjuvants present in the formulates were determined in groundwater in order to determine their leaching potential. Fig. 2 shows a profile in which NP, OP, and BPA were encountered in surface and groundwaters, and the corresponding standard. In this study, in all the sampled fields, pesticide treatments had been carried out during at least 5–10 consecutive years.

The analysis of agricultural aquifers revealed the presence of different types of herbicides, mainly triazines and their degradation products, and chloroacetanilides at levels up to 2.5 µg/l, depending on the application period and rain regime. Fig. 3 shows the total amount of pesticides encountered in the four different well samples. In surface and groundwater samples from the same area, the persistent NP and OP, which are the degradation products of alkylphenol ethoxylates and carboxylates, and BPA, which is widely used as a modifier and stabilization agent in the polymerization of plastics, were detected at levels up to 0.35 µg/l. It has to be stressed that samples with a high concentration of pesticides did not always have correspondingly high concentrations of NP, OP, and BPA. Samples 1, 2, and 3 contained a total pesticide concentration between 1 and 2 µg/l, while in only two cases (sample 1 and 3) surfactants were present. Sample 2, with 1.1 µg/l of total pesticide, contained no NP nor OP, indicating that the origin of pesticide concentration was probably due to diffuse pollution. In well 4, the amount of NP, OP, BPA, and total pesticide was very similar, with relatively high values (between 0.1 and 0.4 µg/l); this can be attributed to the fact that formulations are not the unique source of NP, OP, and BPA in groundwater, but they can also be present as a consequence of sewage sludge

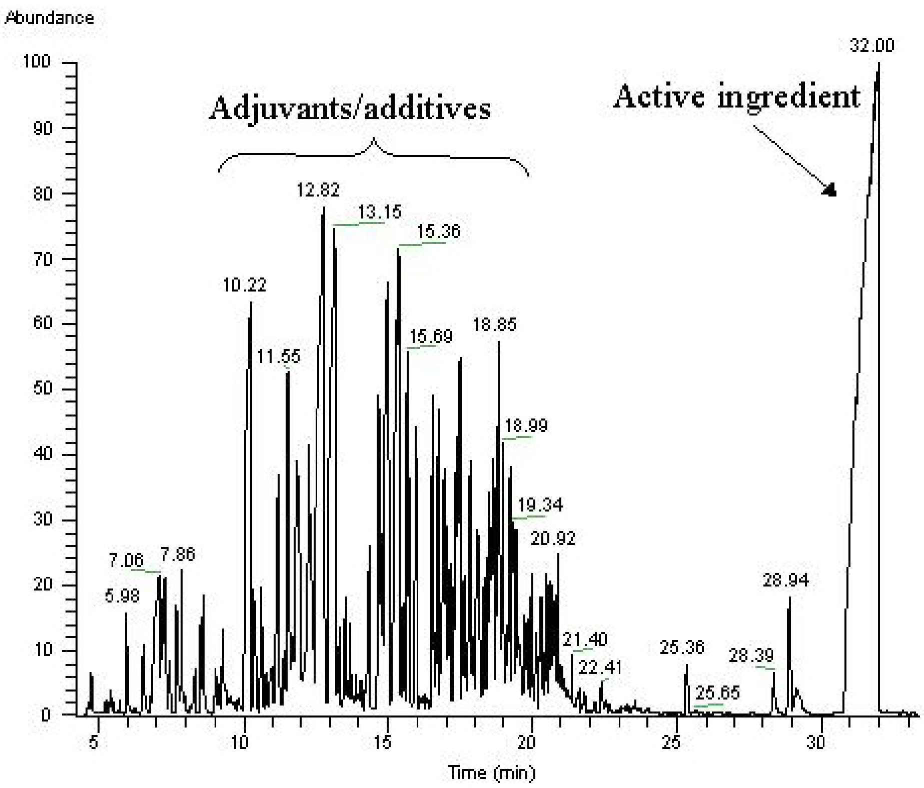

**FIGURE 1.** GC-EI-MS chromatogram of a formulated pesticide in which the active ingredient elutes at 32 min. Peaks from min. 10 to 21 correspond to pesticide adjuvants (surfactants, etc.).

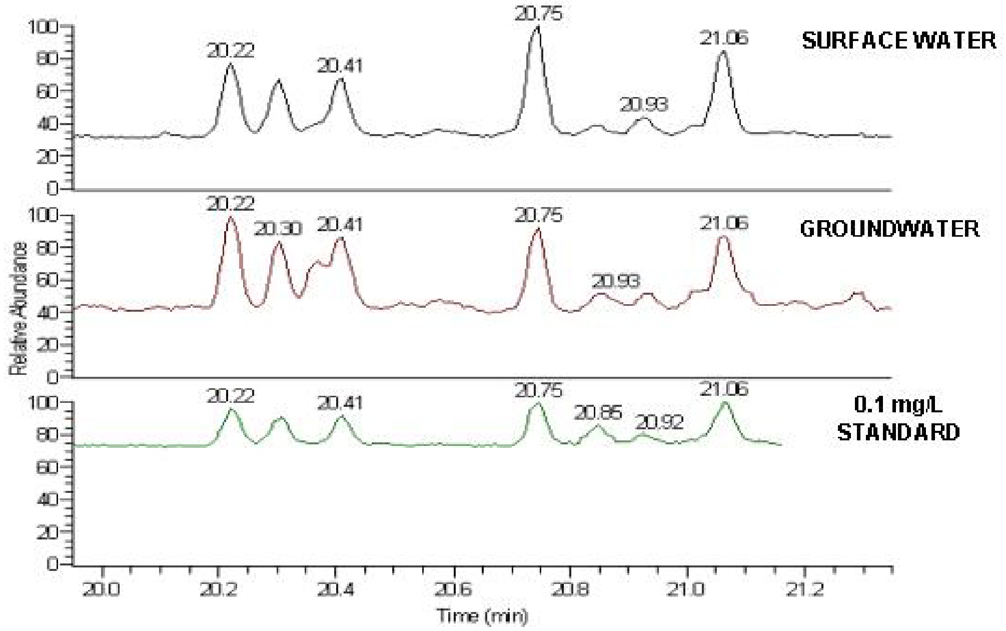

**FIGURE 2.** GC-EI-MS chromatogram of NP, OP, and BPA in surface water, groundwater, and a standard.

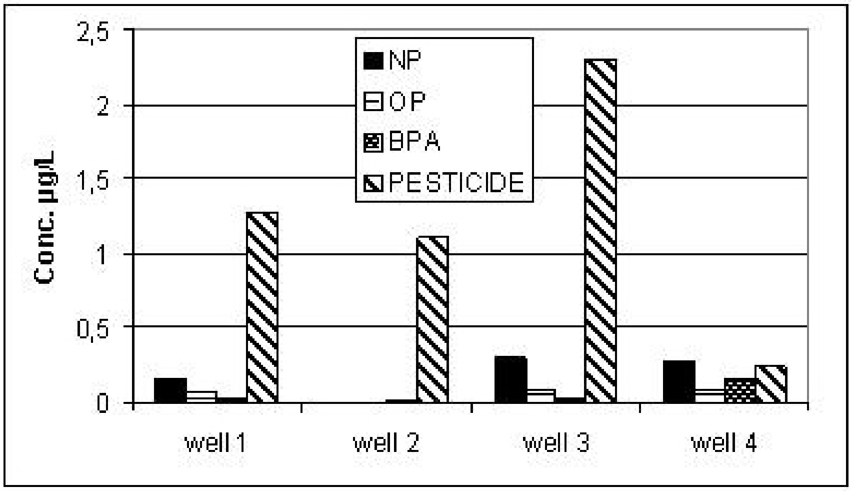

**FIGURE 3.** Concentration (µg/l) of total herbicides, NP, OP, and BPA in four well samples collected in highly agricultural areas of Catalonia.

application in crops. Overall, the presence of such high concentrations of these compounds indicates that groundwater can act as a reservoir of organic pollutants due to the high persistence of these compounds, given by their octanol-water coefficients and low biodegradation rates.

## CONCLUSIONS

As a part of a monitoring program aimed at EDCs in groundwater, an assessment of the presence of formulation additives was performed with the aim of determining their presence in highly agricultural areas. To this purpose, groundwater samples of different confinements were collected from wells of 10–20 m deep and were SPE and analyzed by GC-MS. This analytical technique was apt to determine herbicides as active ingredients of the formulations, NP and OP as a result of the degradation of surfactants used in formulations, and BPA, used in the plastic cans. NP, OP, and BPA were found at levels up to 2.5 µg/l, indicating that pesticide adjuvants are potential and high-risk pollutants that can be found in groundwaters. The presence of all of these compounds represents a substantial decrease in the quality of groundwaters, which are often used for irrigation or in some cases for drinking purposes. Future work will be directed to survey the organic composition of groundwater to identify specific sources, their half lives, and in the last instance, possible remediation actions. This information will serve to determine potential effects towards the environment.
